# Prevalence and Diagnostic Approach to Sleep Apnea in Hemodialysis Patients: A Population Study

**DOI:** 10.1155/2015/103686

**Published:** 2015-07-01

**Authors:** Valentina Forni Ogna, Adam Ogna, Menno Pruijm, Isabelle Bassi, Emilie Zuercher, Georges Halabi, Olivier Phan, Roberto Bullani, Daniel Teta, Thierry Gauthier, Anne Cherpillod, Claudine Mathieu, Alexandra Mihalache, Francoise Cornette, José Haba-Rubio, Michel Burnier, Raphaël Heinzer

**Affiliations:** ^1^Department of Nephrology and Hypertension, University Hospital of Lausanne (CHUV), 1011 Lausanne, Switzerland; ^2^Centre for Investigation and Research in Sleep (CIRS), University Hospital of Lausanne (CHUV), 1011 Lausanne, Switzerland; ^3^Hemodialysis Unit, Northern Vaud Hospital, 1400 Yverdon, Switzerland; ^4^Hemodialysis Unit, Broye Intercantonal Hospital, 1530 Payerne, Switzerland; ^5^Hemodialysis Unit, EHC Hospital, 1110 Morges, Switzerland; ^6^Hemodialysis Unit, Riviera Providence Hospital, 1800 Vevey, Switzerland; ^7^Hemodialysis Unit, Cecil Clinic, 1011 Lausanne, Switzerland

## Abstract

*Background*. Previous observations found a high prevalence of obstructive sleep apnea (OSA) in the hemodialysis population, but the best diagnostic approach remains undefined. We assessed OSA prevalence and performance of available screening tools to propose a specific diagnostic algorithm. *Methods*. 104 patients from 6 Swiss hemodialysis centers underwent polygraphy and completed 3 OSA screening scores: STOP-BANG, Berlin's Questionnaire, and Adjusted Neck Circumference. The OSA predictors were identified on a derivation population and used to develop the diagnostic algorithm, which was validated on an independent population. *Results*. We found 56% OSA prevalence (AHI ≥ 15/h), which was largely underdiagnosed. Screening scores showed poor performance for OSA screening (ROC areas 0.538 [SE 0.093] to 0.655 [SE 0.083]). Age, neck circumference, and time on renal replacement therapy were the best predictors of OSA and were used to develop a screening algorithm, with higher discriminatory performance than classical screening tools (ROC area 0.831 [0.066]). *Conclusions*. Our study confirms the high OSA prevalence and highlights the low diagnosis rate of this treatable cardiovascular risk factor in the hemodialysis population. Considering the poor performance of OSA screening tools, we propose and validate a specific algorithm to identify hemodialysis patients at risk for OSA for whom further sleep investigations should be considered.

## 1. Introduction

Sleep complaints are common in hemodialysis (HD) patients, with a reported prevalence of 50 to 80% [1, 2], including insomnia, sleep apnea (comprising the obstructive and the central form), and restless legs syndrome as the most frequent manifestations.

The reported prevalence of obstructive sleep apnea (OSA) in HD populations is highly variable, ranging from 20 to 80%, owing to differences in the studied populations, diagnostic tools, and OSA definitions [1–7].

Despite these large differences, the prevalence of OSA in HD patients appears to be remarkably higher than in the general population, which has been reported to be 5 to 34% [8–11]. Studies in the general population revealed an important proportion of undiagnosed cases [8–10], which is probably also the case in the HD population.

OSA causes a disruption of sleep, leading to daytime symptoms, such as excessive daytime sleepiness [12, 13]. In the meantime, repeated oxygen saturation drops increase the oxidative stress and stimulate the sympathetic system, leading to hypertension and an increased cardiovascular risk [14–16]. Diagnosing OSA is therefore important in the management of HD patients with high cardiovascular morbidity and mortality, since it is a treatable condition.

The clinical presentation of OSA in HD patients differs from its presentation in patients without end-stage renal disease (ESRD). As such, classical symptoms such as loud snoring seem to be less often present, whereas others (hypertension and fatigue) are common in hemodialysis patients [17, 18]. Usual screening tools for OSA are mainly based on these symptoms and might therefore be less accurate in HD patients than in the general population.

The aims of this study were to assess the prevalence of OSA in a European HD population to evaluate the predictive value of classical screening tools and to develop a specific diagnostic algorithm for HD patients.

## 2. Materials and Methods

### 2.1. Patients

Between June 2012 and June 2013, all the patients attending 6 hemodialysis centers in the western part of Switzerland were offered to be screened for OSA if they fulfilled the inclusion criteria. Participating centers were one university hospital department, 4 hemodialysis units located in peripheral hospitals, and one private dialysis center.

Inclusion criteria were being on chronic intermittent hemodialysis, age ≥18 years, and agreement to participate in the study. Patients were excluded if they had decompensated congestive heart failure or cognitive impairment/active psychiatric disease limiting their ability to understand the questionnaires.

### 2.2. Study Protocol

We conducted a multicenter, cross-sectional population study.

Each participant underwent a nocturnal polygraphy and completed a set of questionnaires.

Anthropometric parameters were measured; patients performed a 24 h urine collection to quantify residual diuresis and underwent a complete laboratory analysis.

The study complied with the Declaration of Helsinki and was approved by the Institutional Ethics Committee (Commission d'éthique de la recherche clinique, Lausanne, Switzerland). All participants provided written informed consent.

### 2.3. Questionnaires and OSA Screening Scores

Patients completed a set of questionnaires during the dialysis session.

Daytime sleepiness was evaluated by the* Epworth Sleepiness Scale (ESS)*, using a cut-off value of >10 points to define excessive daytime sleepiness [12, 19]. The subjective sleep quality item of the Pittsburgh Sleep Quality Index was used to assess sleep quality [20]. Data on sleep medications (hypnotics and benzodiazepines) taken before the sleep recording were collected in the morning.

The screening scores for OSA were Berlin Questionnaire, STOP-BANG score, and Adjusted Neck Circumference.

*Berlin Questionnaire* (BQ) consists of 11 items covering 3 categories (snoring, sleepiness, and hypertension/obesity). Patients are considered at risk for OSA if 2 or more categories are positive [21].

The* STOP-BANG score* consists of 8 items (snoring, tiredness, observed sleep apnea, pressure (hypertension), BMI > 35 kg/m^2^, age > 50 years, neck circumference > 40 cm, and male gender). Each positive item adds 1 point; a score ≥ 3 out of 8 is considered high risk for OSA [22].

The* Adjusted Neck Circumference (ANC)* is calculated by measuring the patient's neck circumference (in cm) and adding additional centimeters if hypertension (4 cm), snoring (3 cm), and nocturnal choking (3 cm) are present. An ANC >48 cm indicates a high probability of OSA [23, 24].

### 2.4. Nocturnal Polygraphy

*Nocturnal Home Polygraphy* (PG) was performed on the night preceding hemodialysis using an Apnea Link Plus device (ResMed Corporation, San Diego, CA, USA) including a chest motion band, a finger pulse oximetry, and a nasal pressure cannula to analyze respiration.

All respiratory events were manually scored by the same experienced pulmonologist (AO) according to the American Academy of Sleep Medicine criteria [25]. The Apnea-Hypopnea Index (AHI) was calculated as the number of apneas and hypopneas per hour of recording, and OSA diagnosis was retained for AHI ≥ 15/h (moderate to severe OSA).

### 2.5. Anthropometric Parameters

*Neck circumference* was measured in the sitting position with a nonstretchable tape above the cricothyroid membrane just before the hemodialysis session following PG recording.

Weight was measured at the end of the hemodialysis session to reflect nutritional status, in light indoor clothing without shoes, using a calibrated Seca scale and height was measured to the nearest centimeter using a wall-mounted stadiometer.

*BMI* was calculated as weight divided by height in square meters. Obesity was defined as a BMI ≥ 30 kg/m^2^ [26].

*Hypertension* was defined as previous diagnosed hypertension or ongoing antihypertensive treatment.

### 2.6. Hemodialysis Characteristics

The hemodialysis schedule was not altered by the participation in the present study. All the patients underwent thrice weekly hemodialysis; HD sessions of the participating centers were performed during the morning or afternoon; there were no evening shifts. The hemodialysis efficacy was assessed using urea kinetic modelling and expressed as equilibrated Kt/V (eKt/V), according to the KDOQI recommendations [27]. Interdialytic weight gain was used to account for the fluid state variation due to HD and computed as the weight difference between beginning and end of the HD session that followed PG.

### 2.7. Statistical Analysis

Statistical analysis was conducted using Stata 11.0 for Windows (StataCorp LP, College Station, TX, USA).* t*-test, chi-square test, and Wilcoxon rank sum test were used to compare the characteristics of the patients with and without OSA. Statistical significance was established at *p* < 0.05.

The performance of the different screening tools to predict the presence of OSA, as diagnosed by PG, was assessed by computing sensitivity, specificity, and positive and negative predictive values.

A study population of 100 subjects was calculated as necessary to detect OSA with 80% sensitivity with 5% alpha error and 10% precision, assuming a 57% prevalence of OSA [7].

ROC analysis was performed to evaluate the overall performance of the screening tests. Subjects with Cheyne-Stokes respiration were not considered in the evaluation of the OSA screening tools.

In order to develop and validate a new diagnostic algorithm for OSA, the patients were divided in a derivation population (all patients from the hemodialysis unit of the University Hospital of Lausanne, the main study center) and an independent validation population (all patients from the other centers).

A predictive logistic regression model was fitted to the derivation set, including all the variables present in classical screening scores: age, gender, BMI, neck circumference, hypertension, daytime sleepiness, snoring, and unrefreshing sleep (Model 1). A second prediction model (Model 2) was fitted using the most significant factors from Model 1 (with *p* < 0.20) and stepwise adding hemodialysis characteristics to maximize both discriminatory accuracy (evaluated by the area under ROC curve) and goodness of fit (Hosmer-Lemeshow test).

The factors identified in Model 2 were entered in a classification and regression tree analysis (CART, a chi-square based nonparametric technique) to evaluate the best discriminatory factors and cut-offs in a classification algorithm of the observations.

Based on the results of the CART analysis, we developed a screening algorithm to identify the patients at risk for OSA and we assessed its performance by computing sensitivity, specificity, positive and negative predictive values, and area under ROC curve in the validation population.

## 3. Results

### 3.1. Study Population

Of the 235 screened candidates, 35 were not eligible and 75 declined to participate.

125 patients were included in the study. 104 subjects (66 men and 38 women) completed home PG and were considered for the final analysis. Reasons for dropout were withdrawal of consent (13), technical problems with the PG recording (3), and loss to follow-up before completing PG (3 patients died, 1 was transplanted, and 1 was transferred to another HD unit).

Patients who completed the study were younger (61.7 ± 14.9  versus  69.2 ± 16.1 years, *p* < 0.01); there was no difference in sex distribution, time on renal replacement therapy, sleep quality, and daytime sleepiness. For all 3 calculated screening scores for OSA, the proportion of positive tests was similar in patients who completed the study and in those who did not.

Demographic, anthropometric, and medical data of the studied population are detailed in Table 1.

### 3.2. Prevalence of Sleep Apnea and Associated Clinical Features

Only 14% (15 patients) had a normal polygraphy (AHI < 5/h); 30% had mild OSA (AHI 5–15/h), 25% had moderate OSA (AHI 15–30/h), and 31% had severe OSA (AHI ≥ 30/h). 11 out of 58 patients (19%) with moderate to severe OSA (AHI ≥ 15/h) had been previously diagnosed and only 6 (10%) were already treated.

Four out of 104 patients (4% of the population) had a central sleep apnea with a Cheyne-Stokes respiratory pattern.

Sleep related symptoms were present in a minority of the study population and did not significantly differ between patients with and without OSA (Table 1). Patients with OSA were older and more often men and had a larger neck circumference. Hypertension was highly prevalent (91%) in the study population but not significantly different between patients with and without OSA.

Among the characteristics of kidney disease and hemodialysis, only time on renal replacement therapy (RRT) differentiated the 2 populations, with OSA patients being on RRT for a longer time.

### 3.3. Performance of the Usual Screening Instruments for OSA

The performances of the 3 existing screening scores are reported in Table 2.

Berlin's Questionnaire (BQ) was positive in 49 patients, classifying them at high risk of having OSA. Only 28 (57%) out of these patients had an AHI ≥ 15/h, corresponding to a positive predictive value (PPV) of 57%.

STOP-BANG score had a higher sensitivity, assigning 67 patients to the group having high risk of OSA. 71% of the patients were correctly classified.

Twenty patients had an Adjusted Neck Circumference (ANC) > 48 cm. This score had the highest specificity of the 3 tests but demonstrated a very poor sensitivity.

When the usual cut-off value of the tests was not considered and all tests were compared using a ROC analysis, the STOP-BANG score and the ANC showed a similar performance, with a ROC area of 0.652 [SE 0.085] and 0.655 [SE 0.083], respectively, whereas Berlin's Questionnaire was less accurate with a ROC area of 0.538 [SE 0.093].

### 3.4. Prediction of OSA in the Hemodialysis Population

The characteristics of the derivation and validation populations are summarized in Supplementary Table S1 in Supplementary Material available online at http://dx.doi.org/10.1155/2015/103686.

In the multivariate model based on the factors of classical screening scores (Model 1), only age, neck circumference, and hypertension were significantly associated with OSA, while BMI and sleep related symptoms showed a nonsignificant association (Table 3). The addition of hemodialysis-related factors (Model 2) improved the discriminatory accuracy and precision of the model.

**Figure 1:**
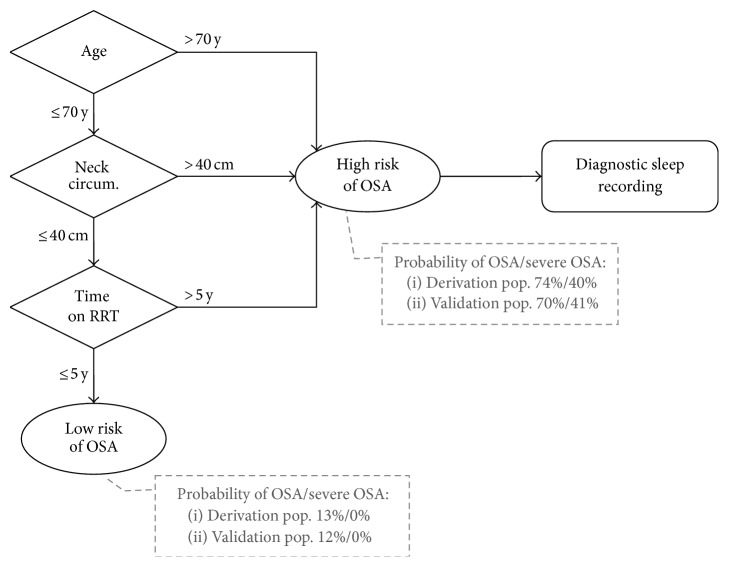
Proposed diagnostic algorithm (ANT algorithm). OSA: obstructive sleep apnea; Neck circum.: neck circumference; RRT: renal replacement therapy. Further investigation by objective sleep recording may be indicated in the low-risk group according to the clinical context (i.e., as a part of a presurgical assessment).

The classification and regression trees (CART) analysis identified age > 70 years, neck circumference > 40 cm, and time on RRT > 5 years as the best discriminatory factors. These factors were used to create a specific screening algorithm: the ANT algorithm (Figure 1).

The application of the ANT algorithm to the validation population led to the categorization of 27 patients (63%) as being at risk for OSA, with higher sensitivity and negative predictive values than the classical screening tools (Table 2). The proposed algorithm had 100% negative predictive value for severe sleep apnea in both the derivation population and the validation population (Figure 1).

**Figure 2:**
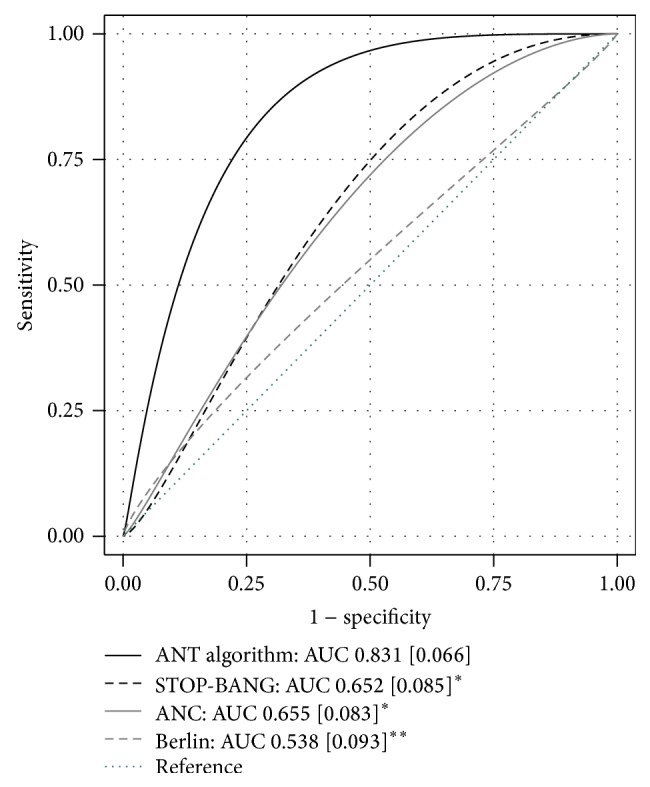
ROC curves of the different screening strategies (validation population). AUC: area under ROC curve [SE]. ^*∗*^ *p* < 0.05 compared to ANT algorithm (^*∗∗*^ *p* < 0.01).

On ROC analysis, the ANT algorithm performed significantly better than the 3 classical screening tools, showing a ROC area of 0.831 [SE 0.066] when applied to the validation population (Figure 2).

## 4. Discussion

This is the largest cohort study in Europe using an objective method (polygraphy) instead of questionnaires to assess the prevalence of OSA in ESRD patients undergoing hemodialysis. The prevalence of moderate to severe OSA in this population was 56%, and 31% of the subjects had severe OSA (with a clear indication for treatment), but only a small proportion of all OSA patients (19%) had been previously diagnosed and even less (10%) were treated.

The usual screening tools for OSA, Berlin's Questionnaire, STOP-BANG score, and Adjusted Neck Circumference, performed poorly in this population and thus appear to be ineffective in daily clinical practice. In this study, we developed and validated a diagnostic algorithm specifically dedicated to hemodialysis patients with a high sensitivity and predictive value to help clinicians screen their patients for OSA.

The largest epidemiologic study in this field reported an OSA prevalence of 23.6% using Berlin's Questionnaire [1], which may have underestimated the prevalence of this condition considering its poor performance in the hemodialysis population. The other trials were small studies with selective recruitment of symptomatic patients [2–5], except for two recent studies performed in USA and Canada in patients with different degrees of chronic kidney disease attending outpatient nephrology clinics [6, 7]. Both studies recruited a subgroup of 75 patients on intermittent hemodialysis, investigated by home polysomnography in the American protocol and by overnight respiratory monitoring in Canada. Our results are in line with the reported prevalence of moderate to severe OSA of 57% in the Canadian study and above the 25.7% prevalence of severe OSA observed in the US study. The lower prevalence in the latter may be due to a less sensitive sensor (thermistor instead of nasal pressure) to assess breathing patterns and to a more restrictive definition of hypopnea requiring a 4% (instead of 3%) oxygen desaturation.

OSA is recognized as an important cardiovascular risk factor in the general population [14, 15]. Although the impact of OSA on clinical outcomes in hemodialysis patients has not yet been evaluated in prospective studies, OSA is likely to contribute to the increased cardiovascular morbidity and mortality observed in this population [28–33]. With such a high prevalence and these potential cardiovascular consequences, OSA screening should be considered as part of the usual workup of ESRD patients, since this condition can be effectively treated with continuous positive airway pressure (CPAP) [14, 34].

For the daily clinical practice, it is important to have a simple tool allowing the patients to be quickly screened, in order to refer those at risk of OSA for further testing. Different OSA screening tools, in the form of questionnaires or scores, have been developed and validated in the general population for this purpose. According to our results, these screening tools are less accurate in the hemodialysis population with poor sensitivity and specificity. For example, Berlin's Questionnaire showed 86% sensitivity and 77% specificity for identifying subjects with AHI > 5/h in the general population [21]. When applied to our hemodialysis population, the same questionnaire performed remarkably worse, with a sensitivity and a specificity just above 50% to identify moderate to severe OSA. Our results are in line with those of Nicholl et al. who demonstrated an accuracy of the 3 screening tools (Berlin's Questionnaire, STOP-BANG, and ANC) ranging from 51 to 67% in the ESRD population [35]. However, they used a less accurate reference test than in our study (simple oximetry instead of polygraphy).

The poor performance of these screening tools in ESRD patients can be explained by the fact that they are mostly based on the typical clinical characteristics associated with OSA, such as excessive daytime sleepiness, unrefreshing sleep, obesity, and hypertension. In our population, the prevalence of these symptoms was not different between subjects with and without OSA, probably because other factors related to renal failure contribute to their development. Our observations support the fact that clinical parameters specific to the hemodialysis population should be considered when screening for OSA in this population. Among the HD related parameters, time on renal replacement therapy emerged as the one with the strongest association with OSA. This effect was independent of age and represents a new finding, suggesting that factors appearing late in the course of the kidney disease could play a role in the increased prevalence of OSA in HD patients. One possible involved mechanism could be chronic fluid overload with overnight fluid displacement to the neck soft tissues increasing upper airway collapse, a phenomenon that has been documented by our research group in hemodialysis patients with OSA [36]. In the present study, neither residual diuresis nor interdialytic weight gain was different between patients with and without OSA, but no direct measurement of fluid overload was performed (i.e., by bioimpedance) and thus we cannot exclude the fact that chronic fluid overload was present in the clinically estimated post-HD “dry” weight. An alternative mechanism could be a dysfunction of upper airway and respiratory muscles secondary to chronic uremic neuropathy or myopathy [37, 38].

To address the limitations of classical screening tools, we propose a simple screening algorithm, based on readily available variables: age, neck circumference, and time on renal replacement therapy. In our validation population, the proposed screening algorithm performed considerably better than usual screening tools, even when the tests were compared by ROC analysis, considering different cut-offs. This simple algorithm could be easily used by clinicians to screen their patients for OSA and select those who need further testing.

There are also limitations in our study that need to be considered. First, although this is the largest study on a hemodialysis population using nocturnal recordings, 31% of the subjects declined to have a sleep recording, which may have induced a selection bias. However, since patients who refused the recording were older than those who accepted and OSA tends to increase with age, this may have led to an underestimation of OSA prevalence. This refusal rate also highlights the fact that sleep recordings, although noninvasive, represent an important burden for hemodialysis patients and the fact that a simple clinical algorithm like the one we propose would be easier to use as a first line screening tool. Second, even though we used independent groups of patients for the creation and the validation of the ANT algorithm, this screening strategy will need to be validated in another population. However, since our population has similar demographic and clinical features (28% of patients with age > 70 years and 32% with a time on RRT > 5 years) to the European hemodialysis population [30, 39, 40], we believe that results of ANT algorithm in other populations would probably be comparable.

Finally, we used ambulatory polygraphy instead of the gold standard in-laboratory polysomnography. Even though polygraphy is a recognized diagnostic tool for OSA, the absence of an objective sleep duration measure by electroencephalogram implies an AHI calculation based on the recording time rather than sleep duration which is shorter. As a consequence, severity and prevalence of OSA could have been underestimated. On the opposite side, since all recordings were performed the night preceding hemodialysis, OSA severity could have been overestimated considering the negative effect of fluid overload on OSA. In effect, we recently showed that OSA is more severe on nights preceding a hemodialysis session in overhydrated patients and is reduced by the correction of fluid overload with hemodialysis [36].

In conclusion, our study confirms the high prevalence of OSA and highlights the low diagnosis and treatment rate of this important cardiovascular risk factor in the hemodialysis population.

Considering the poor performance of classical OSA screening tools, we propose a simple screening algorithm specific to the hemodialysis population to identify patients at risk for OSA who need further testing. This diagnostic approach warrants further prospective validation on a larger population prior to introducing the algorithm into clinical practice.

## Supplementary Material

Supplementary Table S1 summarize the characteristics of the derivation population used for the development of the ANT-algorithm compared to the validation population used for its validation.

## Figures and Tables

**Table 1:** Characteristics of the study population.

	All	Moderate to severe OSA (AHI ≥ 15/h)	No or mild OSA (AHI < 15/h)	*p*
*N*	104	58	46	

*Demographic *				
Age (y)	61.7 (14.9)	65.2 (14.1)	57.3 (14.8)	**0.007**
Male sex (*N*, %)	66 (63.5)	44 (75.9)	22 (47.8)	**0.003**
Ethnicity (*N*, %)				
Caucasian	84 (80.8)	49 (84.5)	35 (76.1)	0.545
Asian	4 (3.8)	2 (3.4)	2 (4.4)	
African	15 (15.4)	7 (12.1)	9 (19.6)	
BMI (kg/m^2^)	26.1 (4.6)	26.6 (4.4)	25.5 (4.8)	0.217
Neck circumference (cm)	40.4 (4.4)	42.0 (3.8)	38.4 (4.3)	**<0.001**
Obesity (BMI ≥ 30 kg/m^2^) (*N*, %)	21 (20.2)	13 (22.4)	8 (17.4)	0.526
Hypertension (*N*, %)	95 (91.4)	54 (93.1)	41 (89.1)	0.474

*Hemodialysis *				
Time on RRT (y) [median–IQR]	2.6 [0.9–6.5]	3.8 [1.2–10.3]	1.9 [0.9–3.8]	**0.026**
Residual diuresis (mL)	505 (751)	386 (563)	687 (953)	0.078
Interdialytic weight gain (kg)	0.97 (1.62)	0.99 (1.66)	0.95 (1.57)	0.908
HD duration per week (h)^#^	11.3 (1.0)	11.5 (0.8)	11.2 (1.2)	0.179
Morning HD shift (*N*, %)	69 (66.4)	41 (70.7)	28 (60.9)	0.293
Hemodiafiltration (*N*, %)^#^	25 (24.0)	12 (20.7)	13 (28.3)	0.369
HD access (*N*, %):				
Fistula	80 (76.9)	43 (74.1)	37 (80.4)	0.449
Catheter	24 (23.1)	15 (25.9)	9 (19.6)	
eKt/V	1.51 (0.33)	1.47 (0.33)	1.56 (0.34)	0.203

*Sleep *				
AHI (number/h) [median–IQR]	17.5 [7.0–40.0]	33.0 [22.0–52.0]	6.5 [4.0–10.0]	**<0.001**
ODI (number/h) [median–IQR]	18.0 [9.0–39.0]	38.0 [20.0–49.0]	9.0 [6.8–13.0]	**<0.001**
Epworth score [median–IQR]	5 [3–8]	5 [2–7]	5 [3–9]	0.247
Excessive daytime sleepiness (*N*, %)	17 (16.4)	7 (12.1)	10 (21.7)	0.185
Sleep time (h) [median–IQR]	7 [6–8]	7 [6–8]	7 [6–8]	0.955
Poor sleep quality (*N*, %)	31 (29.8)	18 (31.0)	13 (28.4)	0.759
Snoring (*N*, %)	57 (54.8)	37 (63.8)	20 (43.5)	**0.039**
Unrefreshing sleep (*N*, %)	35 (33.6)	19 (32.8)	16 (34.8)	0.828
Observed apneas (*N*, %)	7 (6.7)	6 (10.3)	1 (2.2)	0.099
Restless legs syndrome (*N*, %)	19 (19.0)	12 (22.2)	7 (15.2)	0.374
Use of sleep medications (*N*, %)	17 (16.4)	7 (12.1)	10 (21.7)	0.285

*Biology *				
Hemoglobin (g/L)	113.4 (12.3)	113.0 (12.9)	114.0 (11.8)	0.684
Phosphates (mmol/L)	1.49 (0.38)	1.43 (0.37)	1.57 (0.37)	0.072
Creatinine (mg/dL)	7.9 (2.3)	7.7 (2.4)	8.2 (2.2)	0.343
BUN (mg/dL)	54.6 (14.0)	54.0 (14.6)	55.2 (13.7)	0.730
Bicarbonates (mmol/L)	22.4 (2.4)	22.1 (2.7)	22.7 (2.0)	0.277

Values are expressed as mean (SD) if not otherwise specified. IQR: interquartile range.

OSA: obstructive sleep apnea; BMI: body mass index; RRT: renal replacement therapy; HD: hemodialysis; eKt/V: hemodialysis efficacy, assessed using urea kinetic modelling; AHI: apnea/hypopnea index; ODI: oxygen desaturation index; BUN: blood urea nitrogen.

^#^All patients on thrice weekly HD with a synthetic HD membrane (polysulfone or polyethersulfone).

**Table 2:** Performance of the screening instruments for OSA.

Test	Sensitivity	Specificity	PPV	NPV	Accuracy
Berlin's Questionnaire	51.9% [42.1–61.6]	54.4% [44.6–64.1]	57.1% [47.4–66.8]	49.0% [39.2–58.8]	53.0%
STOP-BANG	85.2% [78.2–92.2]	54.4% [44.6–64.1]	68.7% [59.6–77.8]	75.8% [67.4–84.2]	71.0%
Adjusted Neck Circumference	29.6% [20.7–38.6]	91.3% [85.8–96.8]	80.0 [72.2–87.8]	52.5% [42.7–62.3]	58.0%
ANT algorithm^*∗*^	90.5% [81.7–99.2]	63.6% [49.3–78.0]	70.4% [56.7–84.0]	87.5% [77.6–97.4]	76.7%

Values are expressed as mean [95% CI].

PPV: positive predictive value; NPV: negative predictive value.

Accuracy: percentage of correctly classified subjects.

^*∗*^ANT algorithm applied to the validation population only.

**Table 3:** Multivariate logistic models for prediction of obstructive sleep apnea.

Factors	Model 1		Model 2		ROC area	Hosmer-Lemeshow
	OR	95% CI	OR	95% CI		
Gender (f)	0.72^*∗*^	0.12–4.20				
Age (y)	1.10	1.02–1.19	1.12	1.02–1.22		
Neck circumference (cm)	1.65	1.16–2.36	1.63	1.13–2.34		
Hypertension	147	2–11613	379	2–87016		
BMI (kg/m^2^)	0.87^*∗*^	0.68–1.10				
Daytime sleepiness	0.58^*∗*^	0.08–4.32				
Snoring	3.91	0.56–27.2	6.57	0.71–60.71		
Unrefreshing sleep	0.26	0.04–1.78	0.10	0.01–1.11	0.900	0.010
eKt/V			0.08	0.00–14.09	0.927	0.259
Time on RRT			1.06	0.92–1.21	0.927	0.387

Model 1: prediction model with classical risk factors (^*∗*^ *p* > 0.20).

Model 2: specific prediction model: significant factors of Model 1 (*p* < 0.20) + stepwise addition of hemodialysis characteristics to maximize discriminatory accuracy (ROC area) and goodness of fit (Hosmer-Lemeshow test).

OR: Odds Ratio and 95% CI in the final cumulative model.

ROC area: area under the ROC curve of the model when the factor is added.

Hosmer-Lemeshow test: goodness of fit of the model when the factor is added.
