# Supplementary material for: Prevalence and Diagnostic Approach to Sleep Apnea in Hemodialysis Patients: A Population Study
Source: Biomed Res Int. 2015 Jul 1;2015:103686. doi: 10.1155/2015/103686 (PMC4502277; doi:10.1155/2015/103686)
Supplement: Supplementary file 1 — Supplementary Table S1 summarize the characteristics of the derivation population used for the development of the ANT-algorithm compared to the validation population used for its validation. [file 103686.f1.pdf]

Supplementary Table S1: characteristics of the derivation population and the validation population for the ANT-algorithm

	Derivation population	Validation population	p
N	57	43	
AHI (no./h) [median - IQR]	18.0 [6.0 - 35.0]	14.0 [8.0 - 30.0]	0.854
OSA with AHI ≥15/h (N, %)	33 (57.9)	21 (48.8)	0.421
Age (y)	58.2 (16.5)	65.4 (11.8)	0.016
Male Sex (N, %)	38 (66.7)	25 (58.2)	0.409
Race (N, %)			0.005
Caucasian	42 (70.0)	42 (95.4)	
Asian	4 (6.7)	0 (0)	
African	14 (23.3)	2 (4.6)	
BMI (kg/m2)	24.8 (4.0)	27.7 (4.8)	0.002
Neck Circumference (cm)	40.0 (3.7)	40.9 (5.1)	0.323
Time on RRT (y) [median - IQR]	3.3 [0.8 - 7.3]	2.1 [0.9 – 3.8]	0.228

Values are expressed as mean (SD), if not otherwise specified. IQR = interquartile range

AHI = apnea/hypopnea index; OSA= obstructive sleep apnea; BMI = body mass index; RRT = renal replacement therapy
